# Supplementary material for: PlantDeepMeth: A Deep Learning Model for Predicting DNA Methylation States in Plants
Source: Plants (Basel). 2025 Jun 5;14(11):1724. doi: 10.3390/plants14111724 (PMC12157073; doi:10.3390/plants14111724)
Supplement: Supplementary file 1 [file plants-14-01724-s001.zip › plants-3632509-supplementary.pdf]

Supplementary Figures

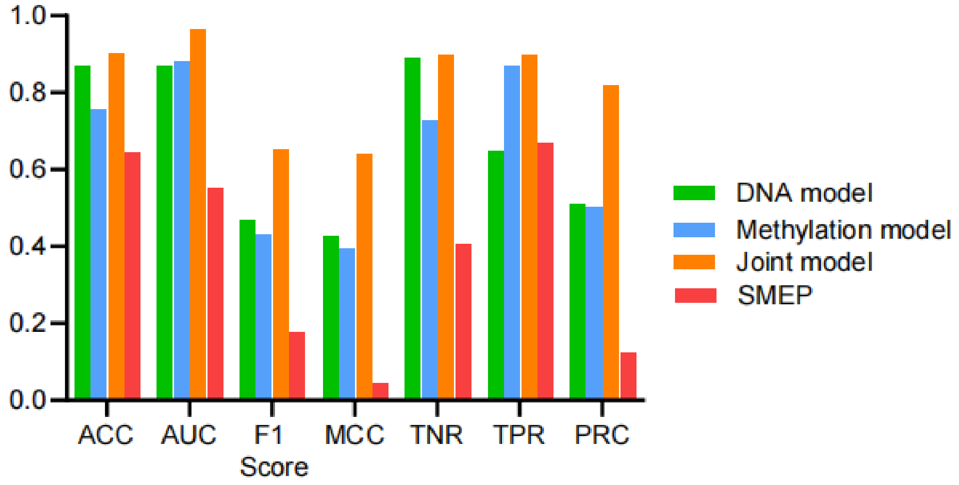

Supplementary Figure S1. The performance evaluation of PlantDeepMeth trained on *Brassica rapa*.

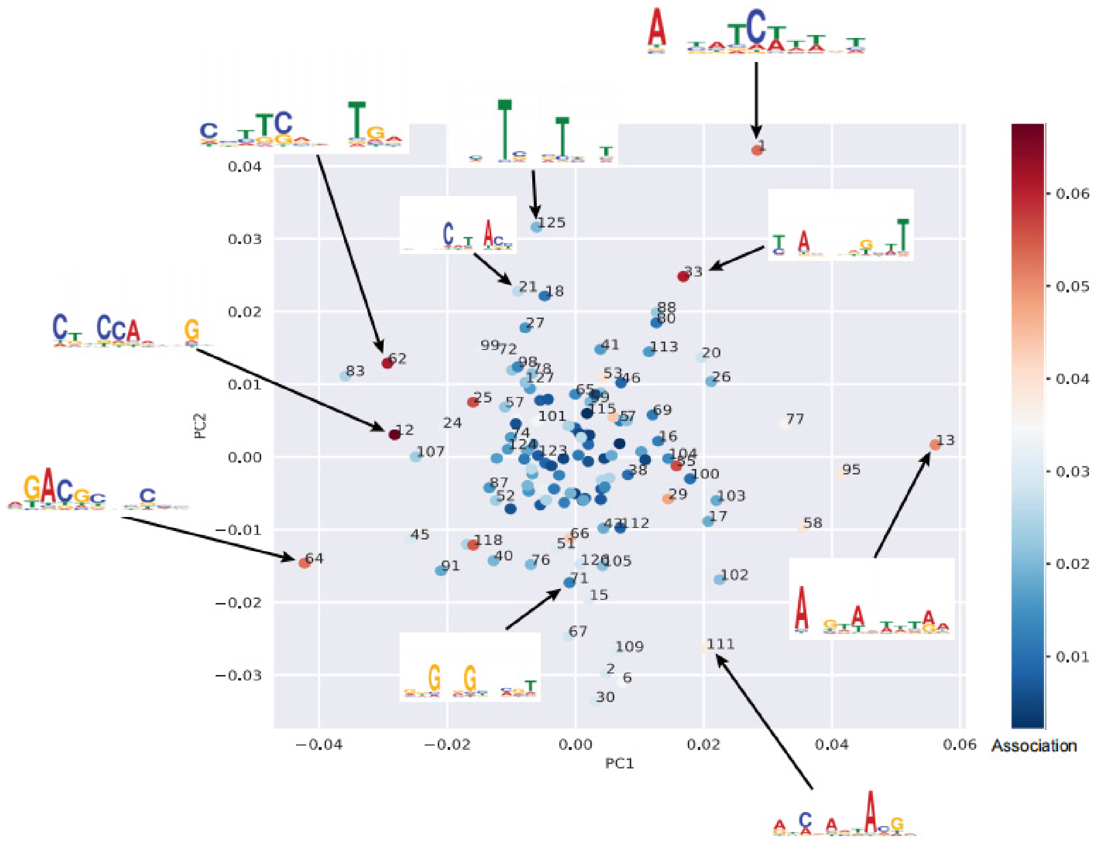

Supplementary Figure S2. Discovered sequence motifs associated with DNA methylation in *Arabidopsis thaliana*. The motifs were identified by PlantDeepMeth after filtering partial sequences. The figure shows the first two principal components of the motif occurrence frequencies in sequence windows (activity). The low to high estimated motif effect on methylation levels is represented by blue to red colors. Sequence logos show the motif related to DNA methylation.

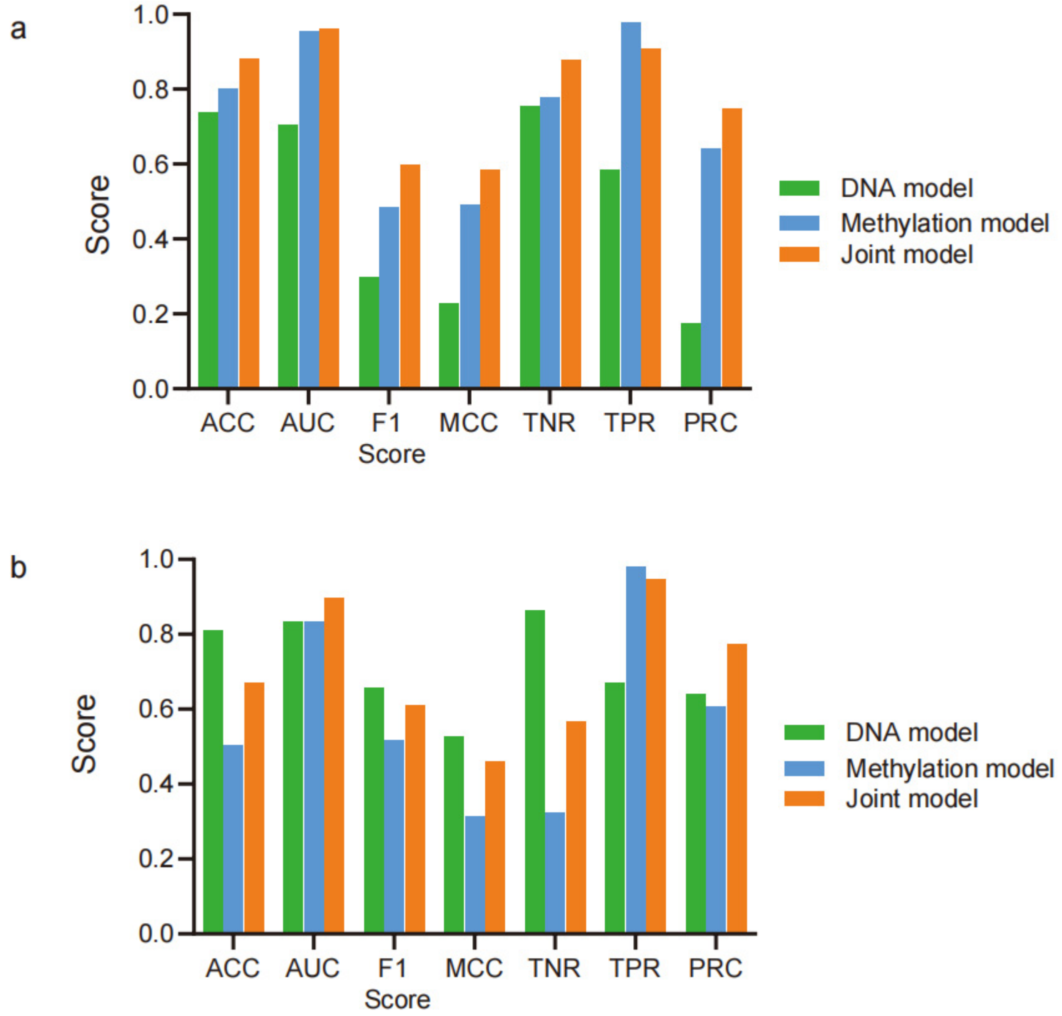

Supplementary Figure S3. Evaluation of generalization ability of PlantDeepMeth based on *Arabidopsis thaliana* training. (a) The test results on *Oryza sativa*. (b) The test results on *Zea mays* embryo.

Supplementary Table

Supplementary Table S1 Cross-species Performance of PlantDeepMeth on *A. thaliana* and *B. rapa*

	train	test	accuracy	AUC	F1 score	MCC	TNR	TPR	PRC
DNA model	*A. thaliana*	*A. thaliana*	0.82	0.81	0.21	0.18	0.84	0.54	0.16
	B. rapa	*A. thaliana*	0.86	0.7	0.16	0.11	0.91	0.28	0.12
	B. rapa	*B. rapa*	0.87	0.87	0.47	0.43	0.89	0.65	0.51
	B. rapa	*A. thaliana*	0.87	0.74	0.39	0.33	0.91	0.46	0.32
Methylation model	*A. thaliana*	*A. thaliana*	0.86	0.93	0.44	0.45	0.84	0.88	0.49
	B. rapa	*A. thaliana*	0.85	0.92	0.41	0.42	0.84	0.84	0.47
	B. rapa	*B. rapa*	0.75	0.88	0.43	0.4	0.73	0.87	0.50
	B. rapa	*A. thaliana*	0.76	0.87	0.44	0.4	0.74	0.87	0.45
Joint model	*A. thaliana*	*A. thaliana*	0.89	0.95	0.53	0.53	0.87	0.91	0.53
	B. rapa	*A. thaliana*	0.92	0.92	0.49	0.45	0.94	0.6	0.42
	B. rapa	*B. rapa*	0.75	0.88	0.43	0.4	0.73	0.87	0.82
	B. rapa	*A. thaliana*	0.76	0.87	0.44	0.4	0.74	0.87	0.63
